# Hole-in-One Mutant Phenotypes Link EGFR/ERK Signaling to Epithelial Tissue Repair in Drosophila

**DOI:** 10.1371/journal.pone.0028349

**Published:** 2011-11-29

**Authors:** Jennifer A. Geiger, Lara Carvalho, Isabel Campos, Ana Catarina Santos, Antonio Jacinto

**Affiliations:** Tissue Morphogenesis and Repair Unit, Instituto de Medicina Molecular, Faculdade de Medicina da Universidade de Lisboa, Lisboa, Portugal; Institute of Science and Technology Austria, Austria

## Abstract

**Background:**

Epithelia act as physical barriers protecting living organisms and their organs from the surrounding environment. Simple epithelial tissues have the capacity to efficiently repair wounds through a resealing mechanism. The known molecular mechanisms underlying this process appear to be conserved in both vertebrates and invertebrates, namely the involvement of the transcription factors Grainy head (Grh) and Fos. In Drosophila, Grh and Fos lead to the activation of wound response genes required for epithelial repair. ERK is upstream of this pathway and known to be one of the first kinases to be activated upon wounding. However, it is still unclear how ERK activation contributes to a proper wound response and which molecular mechanisms regulate its activation.

**Methodology/Principal Findings:**

In a previous screen, we isolated mutants with defects in wound healing. Here, we describe the role of one of these genes, *hole-in-one* (*holn1*), in the wound healing process. Holn1 is a GYF domain containing protein that we found to be required for the activation of several Grh and Fos regulated wound response genes at the wound site. We also provide evidence suggesting that Holn1 may be involved in the Ras/ERK signaling pathway, by acting downstream of ERK. Finally, we show that wound healing requires the function of EGFR and ERK signaling.

**Conclusions/Significance:**

Based on these data, we conclude that *holn1* is a novel gene required for a proper wound healing response. We further propose and discuss a model whereby Holn1 acts downstream of EGFR and ERK signaling in the Grh/Fos mediated wound closure pathway.

## Introduction

Epithelial tissues form a critical barrier between the external environment and an organism's internal organs. All epithelia have developed robust methods for maintaining tissue integrity during natural processes such as cell turnover, as well as restoring barrier function when tissues are damaged. The wound healing process can differ between developmental stages and can also vary among tissues and involve the cooperation of several cell types such as neutrophils and macrophages. One important mechanism, called “purse-string” wound closure, is conserved in epithelial tissues of several animal species including *Drosophila*, chick, mouse, and human [1]. This process involves a rapid assembly of an actomyosin contractile cable in the epithelial cells bordering the wound [2]. Concurrently, these cells extend actin-based protrusions, such as filopodia and lamellipodia, into the wound site. As the contractile cable cinches the wound closed, the wound bordering cells simultaneously elongate in the direction of the wound and contract the edge of the wound. In the final stages of wound closure, lamellipodia and filopodia are required for knitting the wound bordering cells together to form a seamless epithelium. The signaling cascades that regulate the concerted epithelial resealing process as a whole are just beginning to be unraveled. The known molecular mechanisms appear to be conserved in both vertebrates and invertebrates, namely the involvement of the transcription factor Grainy head (Grh) and of the JNK signaling cascade, transduced by AP-1 (Jun/Fos) transcriptional complexes [3]–[7]. In the fly, the expression of some genes at the wound site is dependent on functional Grh and AP-1 binding sites in their promoter region [5], [8]. These observations are consistent with abnormal wound healing in *grh*, *basket/JNK* or *jra/Jun* mutants and activation of JNK signaling pathway at wound sites [3], [5], [9]. The upstream signals activating the cells surrounding the wound are still unknown, but it is established that extracellular signal-regulated kinase (ERK) is phosphorylated upon wounding, an event required at wound sites for a robust closure response [5]. It is also known that ERK can phosphorylate the afore mentioned wound response transcription factors Grh and Fos both in vivo and in vitro [10]–[12]. Furthermore, recent data demonstrated that Stitcher (Stit), a target of Grh transcriptional regulation, encodes a receptor tyrosine kinase (RTK) also capable of inducing ERK phosphorylation in wounded epithelia [13]. All together these data have led to the proposal that a Grh-dependent positive feedback loop likely functions as an amplification mechanism to ensure efficient epidermal wound repair [5], [13].

In a previous screen, we isolated mutants displaying defects in wound healing [9]. One of these identified loci, *CG5198*, is predicted to be involved in processes that are likely associated with wound healing. Specifically, the human homologue of CG5198, CD2 Binding Protein 2 (CD2BP2), binds to the adhesion molecule CD2 and induces cytokine production in T cells, a key component of the mammalian immune response [14], [15]. In a Drosophila cell culture system, CG5198 was found to be involved in the phagocytosis of fungi and bacteria, suggesting a possible role in innate immunity [16]. In other work CD2BP2 has been referred to as U5-52K and is proposed to mediate the assembly of the core spliceosome, a protein complex required for the proper processing of all intron containing RNA transcripts [17]–[19]. As the Drosophila homologue of CD2BP2 was not previously described, we named the *CG5198* locus *hole-in-one* (*holn1*), in honor of the wound healing defect attributed to the mutant *holn1^c07150^*.

In this work we further describe the role of Holn1 in the wound healing process. We reveal the requirement of Holn1 for transcriptional regulation of known ERK/Grh/Fos dependent wound response loci surrounding the wound site. We provide phenotypic evidence suggesting that Holn1 may be involved in other developmental processes requiring Ras signaling. Finally, we analyze the behavior of Epidermal Growth Factor Receptor (EGFR)/ERK signaling mutants and show that reduced EGFR and ERK signaling leads to wound closure defects. We propose a model whereby Holn1 acts downstream of EGFR and ERK signaling in the Grh/Fos mediated wound closure pathway.

## Results

### *holn1* mRNA is expressed ubiquitously in the *Drosophila* embryo and Holn1 protein localizes to the nucleus

**Figure 1:**
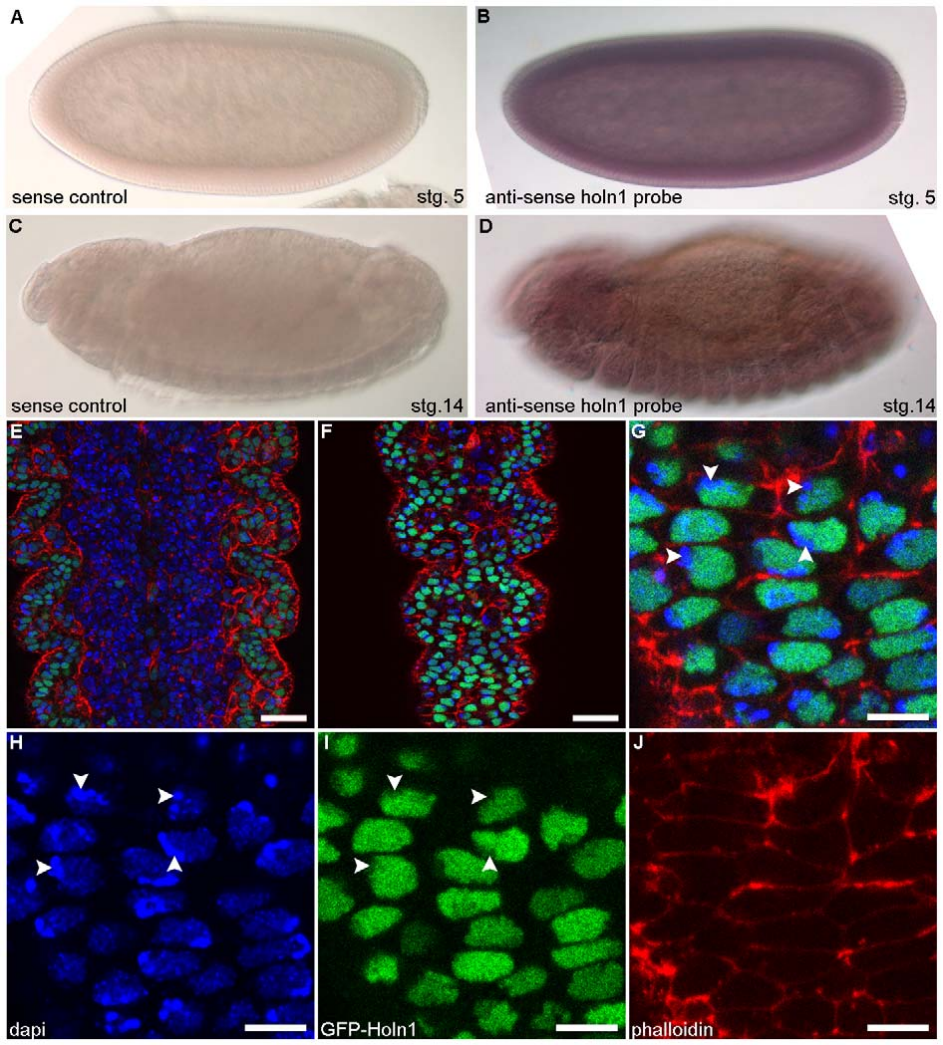
Expression of Holn1 in wild-type embryos. (A–D) Expression pattern of *holn1* RNA in wild-type embryos. (A,C) Sense control in situ hybridization showing lack of staining in stage 5 (A) and stage 14 (C) embryos. (B) *holn1* anti-sense RNA probe shows strong maternal contribution of *holn1* RNA in stage 5 embryo. (D) *holn1* RNA expression is weak and ubiquitous in stage 14 embryo, enriched slightly in the nerve cord and present in the epidermis. Dorsal is to the top and anterior to the left. stg., stage. (E–J) Expression of GFP-Holn1 in the embryonic ventral epidermis under the control of the epidermal driver *e22c>gal4*. (E,F) GFP-Holn1 is expressed in the nuclei of ventral epidermis cells. (G–J) Magnified view of embryo shown in E,F. (E–G) Merged channels. (H) DAPI shows nuclear staining. (I) GFP-Holn1 localization in the cell nuclei. (J) Phalloidin marks filamentous actin at the cell cortex. Arrowheads in (G–I) indicate regions where heterochromatin is more condensed. All images are single Z slices. Scale bar in (E,F) = 20 µm, and in (G–J) = 10 µm.

In situ hybridization of embryos revealed that *holn1* mRNA is maternally deposited (Fig 1A,B) and remains weak and ubiquitous throughout embryonic development (Fig 1C,D). Importantly, *holn1* is expressed in the epidermis at stage 14/15 (Fig 1D), placing it in the right place at the right time to be involved in healing the laser induced wounds implemented in our wounding assay [9]. Expression of GFP-tagged Holn1 (*UAS>GFP-holn1*) using the epidermal driver *e22c>gal4* revealed the nuclear localization of GFP-Holn1 (Fig 1E–J), consistent with the observed distribution of its human homologue CD2BP2 [17], [19], [20]. We noted that GFP-Holn1 signal is reduced in heterochromatin regions, as detected by overlay with areas of intense DAPI staining (see arrowheads in Fig 1G–I).

### *holn1* mutants have wound healing defects

**Figure 2:**
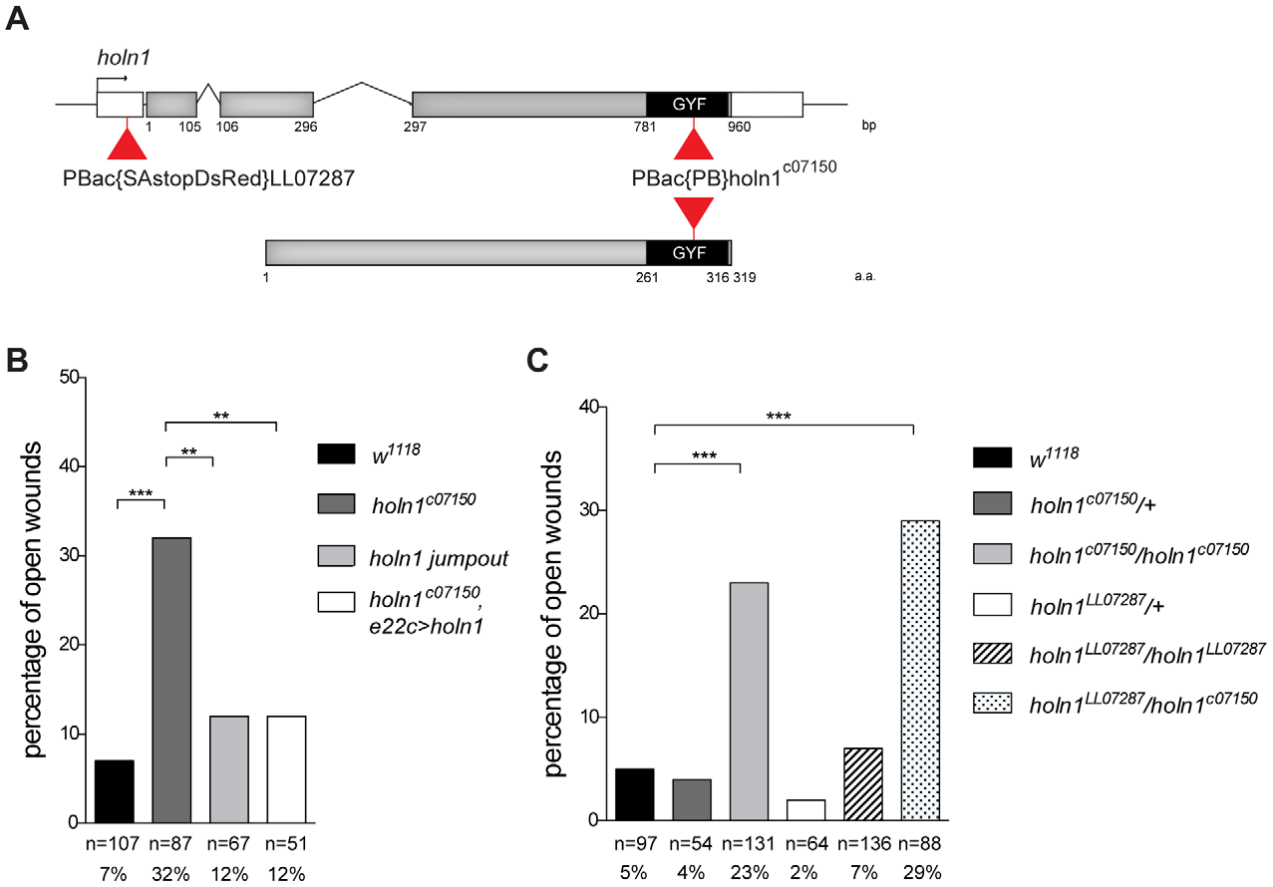
*holn1^c07150^* mutant phenotype is caused by *holn1* loss-function. (A) Gene (upper panel) and protein (lower panel) schematic views of Holn1. UTR's are shown in white and coding regions in grey and black. *holn1^LL07287^* is inserted in the 5′UTR region whereas *holn1^c07150^* is inserted in the third coding exon, in the GYF domain region (black). (B,C) Wound healing phenotypes (% open of open wounds) 16 hours post wounding. (B) Precise excision of *piggyBac* element (*holn1 jumpout*, light grey) and expression of one copy of *holn1* transgene in *holn1^c07150^* mutants (*holn1^c07150^,e22c>holn1*, white bar) restore wound healing defects observed in *holn1^c07150^* mutants (dark grey). (C) *holn1^c07150^/holn1^LL07287^* transheterozygote embryos (spotty bar) show similar percentage of open wounds to *holn1^c07150^* homozygous mutants (light grey bar), in contrast to wild-type (black bar), *holn1^LL07287^* homozygous mutants (striped bar), *holn1^c07150^* heterozygotes (dark grey), and *holn1^LL07287^* heterozygotes (white bar). Fisher's exact test showed significant different between groups (**, p<0.01; ***, p<0.0001).

In our screen [9], we uncovered the wound healing defects of the lethal mutant *holn1^c07150^*, caused by the insertion of a *piggyBac* transposable element after nucleotide 878 of the *holn1* ORF (Fig 2A). This inserted element results in a missense mutation leading to a K to N switch in amino acid position 293, immediately followed by a stop codon likely truncating the C-terminal GYF domain [21]. The GYF domain is the only recognizable functional domain of Holn1 and is characterized as being a protein-protein interacting domain with affinity towards proline-rich regions [22]. In the human Holn1 homologue, the GYF domain is responsible for interactions both with CD2 and with spliceosome components [15], [17], [22]. To confirm that the wound healing defects seen in the *holn1^c07150^* mutant are indeed due to a disruption in Holn1 function caused by the *piggyBac* transposable element, we remobilized this element by precise excision [21]. We observed a complete restoration of wound healing capacity (Fig 2B) and viability (data not shown) upon precise excision of the *piggyBac* element. Also, upon expression of wild-type *holn1* (*pUASp>holn1*) under control of the epidermal driver *e22c>gal4* in *holn1^c07150^* mutant background, we observed a rescue of the wound healing phenotype of *holn1^c07150^* (Fig 2B).

We obtained a second lethal allele of *holn1*, *holn1^LL07287^*, which results from a *piggyBac* insertion in the 5 UTR of the gene [23]. *holn1^LL07287^* fails to complement the lethality of *holn1^c07150^*. We performed the wounding assay on transheterozygous *holn1^c07150^/ holn1^LL07287^* embryos and found a phenotype similar to that of *holn1^c07150^* homozygotes (29% open wounds in transheterozygotes, 23% in *holn1^c07150^* homozygotes, Fig 2C). Interestingly, homozygous *holn1^LL07287^* mutants displayed a weak wound healing defect (7% open wounds, Fig 2C). It is important to note that, in this wounding assay, we were only able to score fully developed hatching larvae for wound healing defects. We observed that 41% of homozygous *holn1^LL07287^* embryos died before hatching, during late embryogenesis, whereas in *holn1^LL07287^/ holn1^c07150^* transheterozygotes and *holn1^c07150^* homozygotes we observed this embryonic lethality phenotype in only 25% and 15% of embryos, respectively (Fig. S1). This suggests that *holn1^LL07287^* might be a stronger allele than *holn1^c07150^* and the early lethality phenotype is dependent on the number of copies of the *holn1^LL07287^* allele.

Taken together, we conclude that *holn1* gene is indeed required for proper wound closure.

### Holn1 is required for efficient wound closure but not for wound edge actomyosin cable formation

**Figure 3:**
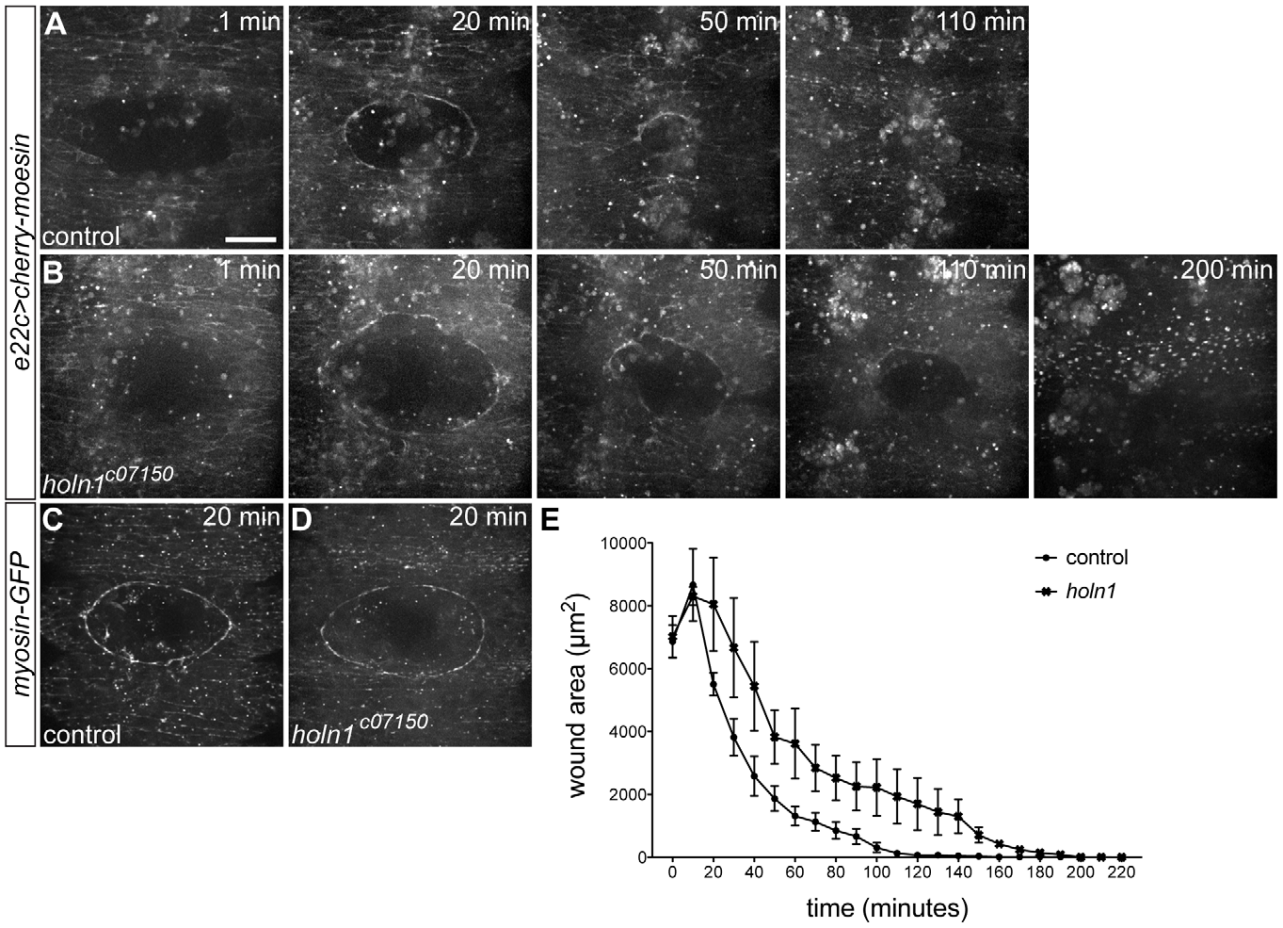
Time course of wound closure in control and *holn1^c07150^* mutant embryos. (A–B) Stills of wound closure process in *e22c>cherry-moesin* control (A) and *e22c>cherry-moesin*,*holn1^c07150^* mutant embryos (B) at 1, 20, 50, 110, and 200 minutes after wounding. At 20 minutes after wounding, an actin cable is already formed in both control (A) and mutant embryo (B). Control embryo has its wound closed at 110 minutes after wounding, whereas mutant embryo only close around 200 minutes after wounding. (C,D) Stills of wounds in *myosin-GFP* control (C) and *myosin-GFP*,*holn1^c07150^* mutant embryos (D) at 20 minutes after wounding. A myosin cable is observed at the wound edge in both control and mutant embryos. (E) Plot showing average wound diameter of closing wounds in control (n = 3) and *holn1^c07150^* mutant embryos (n = 3). Both in control and mutant embryos, wounds start as 7000 µm^2^-diameter holes and expand in size during the first 10 minutes after wounding. After that, wound size decreases exponentially, but slower in mutants than in controls. Error bars represent the standard error of the mean for each time point. Average time of wound closure is significantly higher in mutants (194.3 minutes) compared to controls (127.7 minutes; unpaired t test, p<0.05). Images in (A–D) are projections of spinning disc confocal time-lapse recordings. Scale bar = 20 µm.

To analyze the wound healing phenotype of *holn1* mutants in more detail, we performed time-lapse live recordings of the wound closure process in *holn1^c07150^* mutants, upon laser wounding of the ventral epidermis. We analyzed *holn1^c07150^* mutants, as the *holn1^LL07287^* mutants die earlier making interpretation of the results more difficult. We observed that both control and *holn1^c07150^* mutant embryos assembled a contractile cable containing actin and myosin within minutes upon wounding (Fig 3A–D, Movies S1, S2). Actin-containing cell protrusions also form during wound closure in both cases (see arrows in Movies S1, S2). On the other hand, soon after the actomyosin cable has formed, the wound closure process slows down in *holn1* mutants when compared to controls (Fig 3E). Whereas *holn1* mutant embryos take on average 194 minutes to close 7000 µm-diameter wounds, control embryos take 128 minutes (Fig 3E, n = 3). Together these data indicate that *holn1* is required for efficient wound closure, but not for the immediate assembly of the actomyosin cable.

### *holn1^c07150^* genetically interacts with a constitutively active Ras allele, and RNAi knock-down as well as *holn1^c07150^* phenocopy Ras overactivation phenotypes

**Figure 4:**
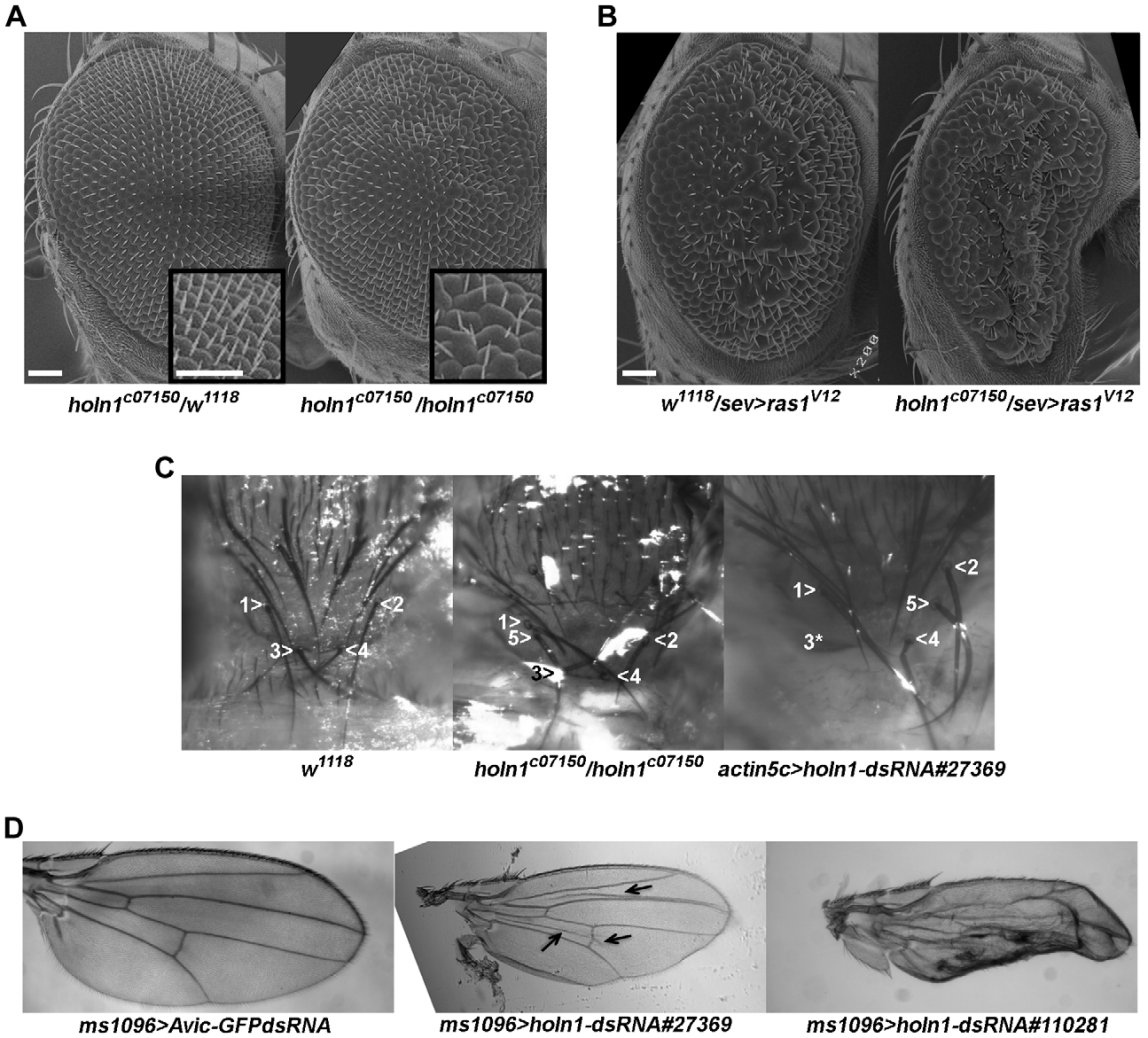
*holn1^c07150^* genetic interaction with *ras1^V12^*, *holn1^c07150^* homozygous escaper phenotypes and RNAi phenotypes in adult tissues. (A) Flies expressing one copy of the *holn1^c07150^* mutant chromosome have eyes with a wild type appearance, i.e. organized lattice of ommatidia (A, left). In contrast, *holn1^c07150^* homozygous escaper flies (A, right) display a weak rough eye phenotype. (A, inserts) Digital zooms of the upper right region of the eye. (B) One copy of the *sev>ras1^V12^* chromosome results in a rough eye phenotype (B, left). This rough eye phenotype is enhanced when combined with one copy of *holn1^c07150^* (B, right). (C) *holn1^c07150^* homozygous escaper (C, middle) and RNAi pupae ubiquitously expressing dsRNA#27369 directed against *holn1* RNA (C, left) have extra and/or misplaced (C, middle and right, #5) and sometimes missing macrochaetae (C, right, 3*) on the scutellum, when compared to wild type flies (C, left). Wild type macrochaetae are arbitrarily labeled 1–4 (C, left) for comparison to the corresponding macrochaetae in mutant flies (C, middle and right panels). (D) RNAi knockdown of *holn1* RNA using the wing specific driver *ms1096>gal4* in combination with 2 independent dsRNAs directed against *holn1*: *UAS>holn1-dsRNA#27369* (D, middle; arrows indicate ectopic veins) and *UAS>holn1-dsRNA#110281* (D, right). Scale bar = 50 µm.

To gain insight on the possible pathways where Holn1 might be playing a role, we analyzed the phenotype of *holn1^c07150^* as well as of *holn1* RNAi knockdown in the adult fly. We were prompted to do this because we observed that rare homozygous *holn1^c07150^* escaper flies (obtained when growing a recombinant stock at 18°C; see Materials and methods) showed clear developmental phenotypes characteristic of EGFR/Ras/ERK pathway mutants. In contrast to heterozygous flies (Fig 4A, left), the *holn1^c07150^* homozygous escapers displayed a subtle rough eye phenotype (Fig 4A, right), reminiscent of the oomatidia fusion phenotype observed in flies expressing a constitutively active form of Ras under the direct control of the *sevenless* (*sev*) eye specific promoter (*sev>ras1^V12^*) (Fig 4B, left) [24]. It is known that EGFR and subsequent Ras activation induce a signaling cascade that is involved in various aspects of organism development, including morphogenesis of the eye, wing and thorax [25], [26]. We performed a classic genetic interaction test and observed that one copy of *holn1^c07150^* dominantly enhanced the *sev>ras1^V12^*-induced rough eye phenotype (Fig 4B, right), when compared to *sev>ras1^V12^* alone (Fig 4B, left). This result indicates that the *holn1^c07150^* mutation causes an increased activation of the Ras signaling pathway, suggesting that wild type Holn1 might function as an inhibitor of this pathway.

Moreover, reducing *holn1* levels by expressing dsRNA [27] directed against *holn1* in the developing wing resulted in a range of phenotypes from wild type looking (less common, not shown) to a smaller, cylindrically curved wing with increased number of veins (Fig 4D, middle), or a blistered wing with much of the surface converted to vein material (Fig 4D, right). In contrast, control wings expressing dsRNA directed against GFP always showed a wild type appearance (Fig 4D, left). EGFR/Ras/ERK signaling is also known to specify the vein regions of the adult wing [26]. The extra vein phenotype observed when knocking down *holn1* using RNAi is also typical of increased EGFR/Ras/ERK signaling during wing development [11], [28], supporting the hypothesis that Holn1 plays a role in this pathway.

Adult thorax macrochaete development is also dependent on the EGFR/Ras/MAPK pathway [25]. We observed that the rare *holn1^c07150^* homozygous escaper flies and late pupae often had extra and/or misplaced or missing macrochaetae on the scutellum (Fig 4C, middle; macrochaete #5 is misplaced or extra), when compared to wild type pupae (Fig 4C, left). We confirmed that this phenotype was due to defects in Holn1 expression by performing RNAi to reduce *holn1* levels in a wild type background. Ubiquitous knockdown of *holn1* using *actin5c>gal4* caused late pupal lethality. We noted that the pupae appeared fragile and fell apart just before hatching, or died during eclosion. When removing the pupal case, we observed a scutellar macrochaete phenotype identical to that seen in the homozygous escapers (Fig 4C, right; macrochaete #3* is missing, and #5 is misplaced/extra). A similar scutellar phenotype was reported when overexpressing EGFR using *apterous>gal4*, although the increase in macrochaete number was ubiquitous throughout the thorax in those experiments [25].

Taken together, these data are consistent with Holn1 involvement in the EGFR/Ras/ERK signaling pathway.

### *holn1^c07150^* mutants show reduced activation of wound reporter genes downstream of wound healing transcriptional pathways

**Figure 5:**
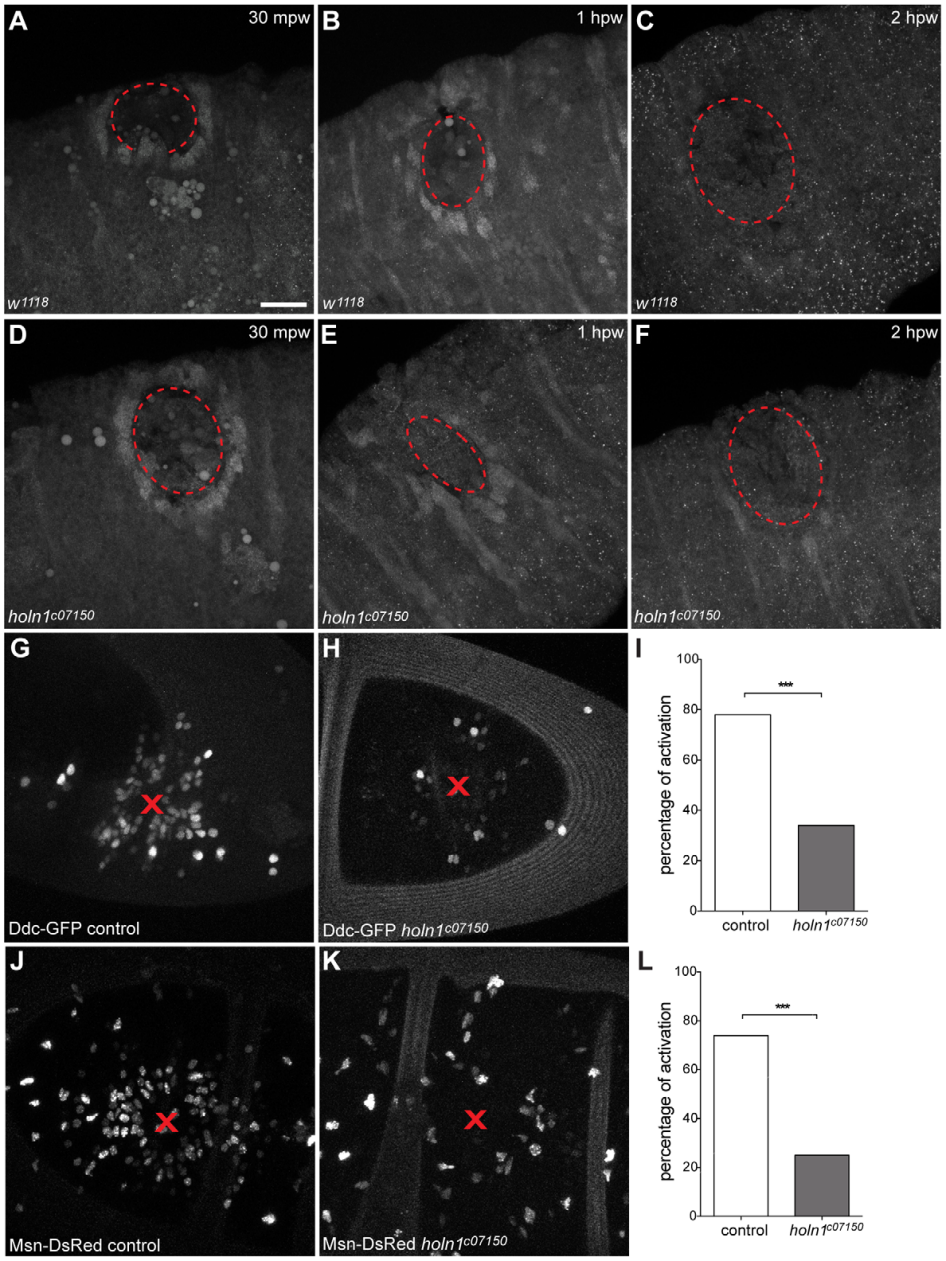
ERK and wound reporter activation in wounded control and *holn1^c07150^* mutant embryos. (A–F) dpERK immunostaining in ventral epidermis of wounded wild-type (A–C) and *holn1^c07150^* mutant embryos (D–F) 30 minutes (A,D), 1 hour (B,E), and 2 hours after wounding (C,F). In both controls and mutants, dpERK is activated in cells surrounding the wound (dashed line) 30 minutes and 1 hour after wounding, and it disappears 2 hours after wounding. (G,H) Ddc-GFP activation in cells around wound region (red arrows) in control (G) and mutant (H) embryos. (I) The number of embryos that activate the wound reporter Ddc-GFP 5 hours after wounding is significantly lower in mutants (34% of embryos, n = 44) compared to controls (78% of embryos, n = 50; p<0.0001). (J,K) Msn-DsRed activation in cells around wound region (red arrows) in control (J) and mutant (K) embryos. (L) The number of embryos that activate the wound reporter Msn-DsRed 5 hours after wounding is significantly lower in mutants (25% of embryos, n = 125) compared to controls (74% of embryos, n = 27; p<0.0001). Dashed lines in (A–F), wound edge; mpw, minutes post wounding; hpw, hours post wounding. Red cross in (G,H,J,K), wound site. Scale bar = 20 µm, except in (J) = 18 µm.

ERK phosphorylation occurs downstream of RTK and Ras activation [29]. It has been shown that phosphorylation of ERK occurs at wound sites in the Drosophila embryo as well as in cell culture systems [5], [30]. As we observed a genetic interaction between Holn1 and Ras signaling, we asked whether Holn1 would have an influence on ERK phosphorylation around the wound site in our system. We observed that, as previously shown [5], [13], ERK appears to be strongly activated in the cells immediately surrounding the wound, as shown by the detection of the diphosphorylated form of ERK (dpERK) by immunostaining (Fig 5A–F). This activation was observed as early as 15 minutes after wounding (data not shown), reaching maximum levels 30 minutes after wounding (Fig 5A). One hour after wounding, ERK phosphorylation decreased around the wound edge and was undetectable two hours after wounding (Fig 5B,C). Surprisingly, we observed a similar pattern of ERK activation in *holn1^c07150^* mutants when compared to wild type (Fig 5D–F). This shows that Holn1 is not required for ERK phosphorylation at the wound edge epithelium, suggesting that Holn1 functions in parallel to or downstream of ERK activation.

It has been recently reported that phosphorylation of ERK upon wounding occurs upstream of a transcriptional activation pathway involved in epidermal wound repair [5], [10]. Particularly, the transcription factors Grh and Fos have been shown to act downstream of ERK and to induce the transcription of genes required for cuticle repair [5], [8]. One of the Grh/Fos target genes, *dopa decarboxylase* (*Ddc*), encodes an enzyme involved in the production of highly reactive quinones involved in crosslinking chitin and cuticle proteins during the construction and repair of the cuticular barrier [31]. Another factor induced by ERK, Grh and Fos upon wounding is the kinase *misshapen* (*msn*) [8]. To determine whether Holn1 is involved in the induction of wound reporter genes downstream of ERK, we compared activation of *Ddc* and *msn* in control and *holn1^c07150^* mutant embryos by using previously described tagged reporters for these genes [5], [8]. In control embryos we observed activation of *Ddc-GFP* and *msn-DsRed* 5 hours after wounding as previously reported, whereas in *holn1^c07150^* mutants the activation of *Ddc-GFP* and *msn-DsRed* was significantly decreased (Fig 5G–L). These observations suggest that Holn1 is required for activation of Ddc and Msn upon wounding. Together, these data suggest that Holn1 might act downstream of ERK in the regulation of Ddc and Msn transcription to promote wound healing.

### EGFR/ERK signaling regulates wound healing

**Figure 6:**
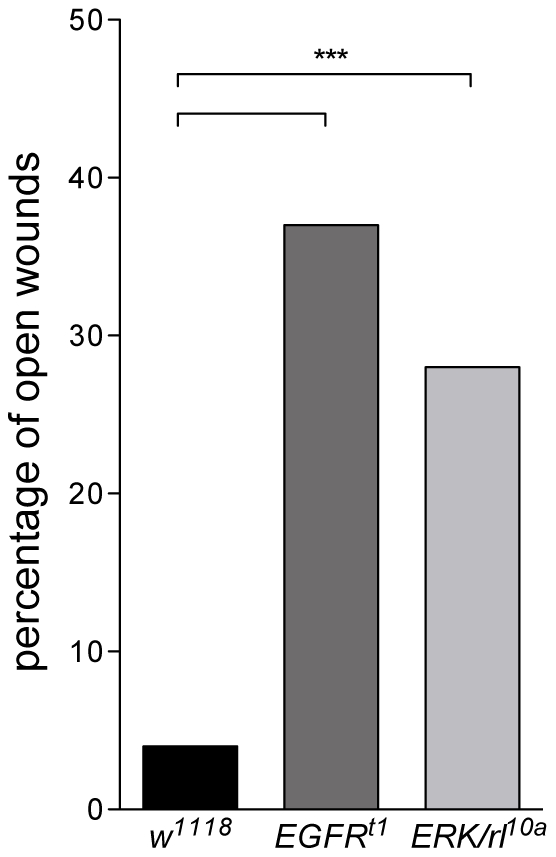
EGFR and ERK are required for wound healing. *EGFR^t1^* and *ERK/rl^10a^* mutants show a significantly higher percentage of open wounds 16 hours post wounding (*EGFR^t1^*, 37%; *ERK/rl^10a^*, 28%) compared to wild-type (*w^1118^*, 5%). n[*w^1118^*] = 138; n[*EGFR^t1^*] = 70; n[*ERK/rl^10a^*] = 71. Fisher's exact test showed significant different between groups (*** = p<0.0001).

Considering that Holn1 appears to be involved in Ras signaling in adult epithelia and that it influences the transcription of genes known to be transcribed downstream of ERK activation, we asked whether the ERK pathway itself was also required during epithelial wound healing. To this end, we analyzed the wound healing phenotypes of *EGFR^t1^* and *ERK/rolled(rl)^10a^* homozygous mutant embryos. *EGFR^t1^* is a homozygous viable hypomorphic mutant for EGFR [32] whereas *ERK/rl^10a^* is described as a strong loss-of-function allele of *ERK/rl* [33]. We found that both mutants have wound closure defects, where *EGFR^t1^* show 37% open wounds and *ERK/rl^10a^* 28% (Fig 6). These data indicate that activation of EGFR/ERK signaling is necessary for proper wound healing to occur.

## Discussion

This work describes the novel gene *holn1* and reveals its involvement during embryonic wound healing. We have found that Holn1 is acting downstream of ERK activation in the ERK/Grainy head pathway when activated during the wound healing process.

### *holn1^c07150^* is a loss-of-function allele of Holn1

The *holn1^c07150^* allele is an insertion of a *piggyBac* element in the coding region of the *holn1* gene [21]. This inserted sequence putatively leads to the production of a premature stop codon within the GYF domain region, shown to be key to the known function of the human homologue of this protein, CD2BP2 [15], [17], [22]. We showed that the lethality and wound healing defects in *holn1^c07150^* could be rescued by ubiquitous expression of *pUASp-holn1* wild-type construct, as well as by precise excision of the *piggyBac* element. Furthermore, we observed that *holn1^ c07150^* heterozygotes do not show wound healing defects or other mutant phenotypes, indicating that this is not a dominant mutation. Together, these results suggest that this allele is a loss-of-function mutation.

The second *holn1* allele, *holn1^LL07287^*, results from a *piggyBac* element insertion in the 5′ UTR region of the gene [23], fails to complement *holn1^c07150^*, and therefore possibly leads to a loss-of-function mutation as well. We observed that *holn1^LL07287^* homozygous mutants have two fold higher percentage of dead embryos compared to *holn1^c07150^*, making *holn1^LL07287^* a stronger allele. The percentage of dead embryos in *holn1^LL07287^* homozygotes and *holn1^LL07287^/holn1^c07150^* transheterozygotes embryos is the same, suggesting that the increase in lethality is independent of genetic background effects. Interestingly, surviving *holn1^LL07287^* homozygote embryos do not display wound healing defects. We believe that these surviving embryos are likely to have developed a mechanism to compensate for Holn1 loss. Although beyond the scope of this work, experiments analyzing Holn1 protein levels in the different mutants would help to further characterize each allele.

### Holn1 is involved in the Ras signaling pathway during eye development and is required for proper thorax and wing development in adult flies

This study reveals that the *holn1^c07150^* allele genetically interacts with a key component of the EGFR/Ras/ERK eye development pathway in the adult fly. In particular, we found that the *holn1^c07150^* mutation enhances the rough eye phenotype induced by constitutively active Ras (Ras1^V12^). This suggests that wild type Holn1 might normally be a suppressor of the EGFR/Ras/ERK pathway. In support of a role of Holn1 in eye development, an analogous, albeit much weaker rough eye phenotype was observed in *holn1^c07150^* homozygous escaper flies.

We provide further phenotypic evidence that Holn1 is involved in additional developmental processes known to be regulated by the EGFR/Ras/ERK pathway. Namely, the phenotypes observed in the adult wing and late pupal/adult thorax tissues resulting from either *holn1^c07150^* mutant or *holn1* RNAi knockdown phenocopy those previously observed upon overactivation of the EGFR/Ras/ERK pathway (Fig 4C,D) [11], [25], [28]. Together, these data suggest that Holn1 might be a suppressor of the EGFR/Ras/ERK pathway during adult development.

These results provided a connection between a gene required for Drosophila wound healing and the EGFR mediated signaling pathway, which prompted us to test whether this pathway is also required during the wound closure.

### EGFR/ERK signaling is required for proper wound healing

Loss of Holn1 function in the *holn1^c07150^* mutants, as well as reduced ERK signaling in *EGFR^t1^* and *ERK/rl^10a^* mutants, impaired the wound healing process. Moreover, ERK activation was detected shortly after wounding and maintained at least until one hour after wounding (Mace, et al. 2005, this study). Although previous studies have proposed that ERK activation is required for proper wound healing [5], [13], direct evidence for this has never been provided. Mace and co-workers have shown that inhibiting ERK phosphorylation by injecting a drug against MAP kinase kinase (MEK) into the perivitelline space of embryos leads to reduced activation of the wound reporter gene *Ddc*, whereas the wound closure at the cellular level has not been addressed [5]. In another study, it has been shown that ERK phosphorylation during wound healing is partially dependent on the RTK Stit, although other RTK(s) must be also involved, as ERK phosphorylation still occurs in the *stit* mutant [13].

**Figure 7:**
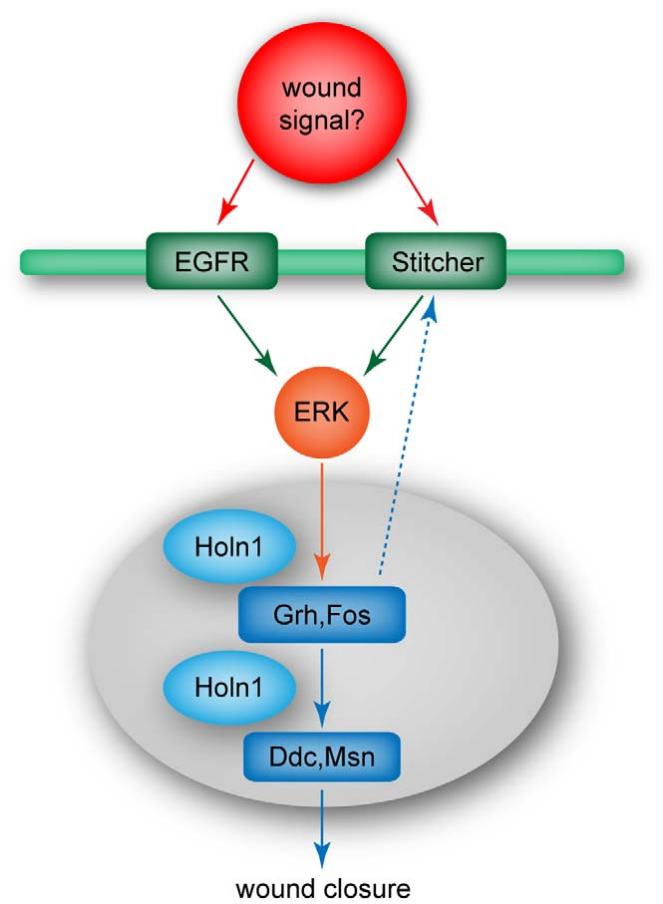
Schematic model of proposed Holn1 function during wound healing. Several signals are generated upon wounding, which activate different RTKs at the cell membrane, such as EGFR and Stitcher. These RTKs activate the ERK pathway leading to activation of Grh and Fos transcription factors, which subsequently induce the expression of cuticle repair genes (Ddc and Msn). One hypothesis for Holn1 function is that Holn1 is involved in the transcription and/or splicing of components of the ERK/Grh/Fos pathway in the nucleus and thereby contribute to proper wound healing.

Our work shows for the first time that both activation of the RTK EGFR, as well as the activation of ERK, canonically found downstream of EGFR signaling [34], are required during wound healing in Drosophila (Fig 7). Further experiments will be necessary to determine whether additional RTKs are involved in wound closure. For instance, it would be interesting to see the effect of a simultaneous knockdown of Stit and EGFR function on ERK phosphorylation and wound closure.

### Holn1 acts downstream or in a parallel pathway to ERK during wound healing

Interestingly, we observed that *holn1* mutants showed similar levels of ERK activation to wild-type embryos upon wounding. This result leads us to propose that Holn1 functions as an activator of the pathway downstream of, or in parallel to, EGFR/ERK signaling during wound healing. In contrast, Holn1 appears to have repressor activity during adult fly development. Indeed, the activity of the Ras pathway is known to have distinct outcomes depending both on levels of Ras signaling as well as on the tissue context [26], [35].

In addition, we observed that loss of Holn1 function results in reduced activation of known wound reporter genes, *Ddc* and *msn*, both previously described to be transcribed downstream of ERK activation and of Grh and Fos transcription factors upon wounding. Grh phosphorylation by ERK was recently shown to be required for Ddc and Msn activation and for the re-establishment of an epithelial barrier after injury [10]. Furthermore, Fos is known to be phosphorylated and activated by ERK during wing vein patterning and neuronal differentiation [11]. Therefore, Holn1 is probably acting in the ERK/Grh/Fos pathway upstream of Ddc and Msn and downstream of ERK during wound healing (Fig 7). This is in agreement with the nuclear localization of GFP-Holn1 protein and its predicted role in mRNA splicing. In the canonical EGFR/Ras/ERK pathway, the activated form of ERK translocates to the nucleus and phosphorylates its targets, thereby inducing gene expression [34]. Human and yeast homologues of Holn1 are known to associate with spliceosome components during early stages of spliceosome assembly [19], [17]. The initial response to wounding appears to involve the transcription of several genes [5], [6]. It is therefore conceivable that Holn1 contributes to efficient wound healing by acting in the nucleus to promote the splicing of components involved in the ERK/Grh/Fos pathway. Consistent with an important role of mRNA splicing in regulating the ERK pathway, a recent report has shown that the exon junction complex (EJC) is required for the splicing of specific introns in the *ERK/rl* gene, and that knockdown of components of the EJC lead to an overall reduction in ERK expression [36]. We show that Holn1 knockdown does not seem to affect ERK phosphorylation levels indicating that Holn1 probably does not play an essential role in ERK splicing. However, it is possible that our immunostaining assay is not sensitive enough to detect a subtle change in ERK protein levels. To clarify this, it would be necessary to use a quantitative method to measure ERK expression levels, such as real time PCR. It has also been shown that different splicing variants of Grh are expressed during embryonic development [37]. Holn1 could also be involved in the splicing of this transcription factor during wound healing and during development in general, yielding *holn1* mutant embryos without certain Grh isoforms that may be required for a rapid response. Another hypothesis is that Holn1 works in a distinct pathway that acts in parallel to the ERK/Grh/Fos pathway. One could begin to address this challenging question by testing if *holn1* mutations affect the expression levels and/or splicing patterns of known components of the ERK/Grh/Fos pathway. Another possible approach would be to determine Holn1 protein-protein interaction partners in the Drosophila embryo using tagged Holn1 to immunoprecipitate associated proteins and determine their identity with mass spectrometry analysis.

We have shown that Holn1 is not required for the initial rapid response to wound infliction, i.e. the formation of the actomyosin cable within minutes of wounding (this work and [2]) and the phosphorylation of ERK, which is also detectable soon after wounding (this work and [5]). This observation is consistent with Holn1 playing an indirect role in the mechanics of wound closure by regulating the mRNA levels of genes required for this process, such as those involved in rapid and productive cable contraction. Interestingly, the actin cable was present in all the wound closure mutants isolated in our previous screen that we have analyzed in more detail, suggesting that regulatory events downstream of cable formation dominate the wound closure process [9]. In any case, it is clear that Holn1 is required to perform some additional function needed to sustain the closure process, as *holn1* mutants take on average 1.5 times longer to close a wound compared to wild type embryos. A similar delay in wound closure was previously reported for *rho1* GTPase mutants, which do not form an actin cable, but can still close small wounds, albeit 2 times slower than wild type embryos [2]. Aside from its possible role in the epithelial hole closure process, Holn1 could also be involved in cuticle repair. As mentioned above, Grh and ERK activity are required for the re-establishment of the epithelial permeability barrier after injury [10]. Thus, Holn1 might be involved in this process by regulating the ERK/Grh pathway.

In the future, just as the *holn1^c07150^* mutation uncovered a connection with the EGF/Ras/ERK signaling pathway and wound healing, microarray analysis of wounded *holn1^c07150^* embryos would identify genes that are likely activated downstream of this wound closure pathway. Performing the same experiment using an alternative splicing array as in [38] would further reveal if Holn1 plays a role in wound dependent splicing events.

## Materials and Methods

### Fly strains and genetics

Crosses were performed at 25°C on standard medium. The *w^1118^* strain was used as control. All strains used were purchased from Bloomington Stock Center (Indiana, USA) unless stated otherwise. *piggyBac(PB)CG5198^c07150^/CyO* and *piggyBac(SAstopDsRed)LL07287/CyO* (Drosophila Genetic Resource Center, Kyoto; [23], renamed respectively *holn1^c0715^* ^0^ and *holn1^LL07287^* in this work, were rebalanced with CyO-CTG.

Remobilization of the transposable element present in *holn1^c07150^* was performed as previously described [21]. The revertant line was confirmed by PCR and sequencing, and homozygous flies were viable.

The following recombinants and stocks were generated using standard methods: 1) For the rescue experiments, *holn1^c07150^,e22c>gal4/CTG; holn1^c07150^,pUASp>holn1(6M)/CTG; holn1^c07150^/CyO; da>gal4/TM6b.* 2) For genetic interaction tests: *sev>Ras1.V12*. 3) For Gal4 expression: *actin5C>gal4/TM6b* (strong ubiquitous expression), *e22c>gal4* (ubiquitous expression starting at stage 12, stronger in the ectoderm), *ms1096>gal4* (wing disc/adult wing), *da>gal4* (moderate ubiquitous expression). 4) For live reporter lines: *UAS>cherry-moesin/CTG* to label actin [39], *sqh-GFP42* to label myosin [40], and *Ddc-GFP* and *Msn-DsRed* for wound reporters [5], [8]. *UAS>cherry-moesin/CTG*, *sqh-GFP42* and *Msn-DsRed* transgenes were recombined with *holn1^c07150^* mutation and for *Ddc-GFP* the *holn1^c07150^/CTG; Ddc-GFP* stock was generated.

The homozygous escaper fly eyes depicted in figure 4A arise from recombinant stock *holn1^c07150^, P(neoFRT)40A* when grown at 18°C, generated using standard methods. The *ERK/rl^10a^* allele was kindly provided by E. Hafen.

### Generation of Holn1 transgenics

To generate *pUASp>holn1* rescue construct/transgenic flies, cDNA of *holn1* coding region was amplified using Pfu Taq polymerase (Promega) from plasmid GH13760 (DGRC) using primers: forward = TCctcgagCTTGATAAAATGGCGAGCAAAAG (adding a Xho1 site at the 5′end) and reverse = TCtctagaCTACAAGTACAAATCGAAATCTATG (adding an Xba1 site at the 3′end). This fragment was cloned into Topo vector pCR2.1 (Invitrogen) and then subcloned into pUASp vector utilizing XbaI and NotI restriction sites. Colonies were tested by PCR using the above primers. One colony was selected and presence of CG5198 insert was confirmed with restriction digest. This construct was used to obtain transgenic flies (BestGene Inc, USA). The obtained homozygous viable fly strain *pUASp>holn1 (6M)* was used for recombination with the mutant chromosome *holn1^c07150^*.

The GFP-Holn1 construct was created using the Gateway® Technology with Clonase™ II (Invitrogen). *holn1 att*B-flanked DNA was amplified from plasmid GH13760 (DGRC) using primers For = ggggacaagtttgtacaaaaaagcaggcttcatggcgagcaaaagaaagc and rev = ggggacccatttgtacaagaaagctggctcctacaagtacaaatcg and recombined into an *att*P-containing donor vector to generate an entry *holn1* clone, using BP Clonase™ II (Invitrogen). This entry clone was next recombined with pPGW destination vector from the Drosophila Gateway^TM^ Vector Collection developed by the Murphy lab, using LR Clonase™ II (Invitrogen). The resulting construct with the pUASp promoter sequence and GFP tag sequence directly upstream of the *holn1* start codon was sequence verified and sent to BestGene Inc (USA) for injection. Transformants were selected by eye color and GFP expression.

### Transgenic RNAi

Transgenic fly stocks containing Gal4 inducible inverted repeat constructs specifically targeting *holn1* were obtained from the Vienna Drosophila Research Center (VDRC), stocks 27369, 27370 (not shown, results identical to 27369, although slightly less penetrant), and 110281**.** All three lines used gave similar results, have no predicted off target hits and are pupal lethal when expressed using *actin5c>gal4* [27] (VDRC and this work). As a control we used a transgenic fly expressing an inverted repeat construct directed against GFP (*pUAS>Avic-GFP.dsRNA.R*). Expression of this construct ablates expression of NLS-GFP at both 25°C and 29°C (S. Prag, unpublished observations). Flies homozygous for the RNAi constructs were crossed to flies expressing *ms1096>gal4* or *actin5c>gal4/TM3-TTG* and selected against GFP and Sb for analysis. All RNAi crosses were performed at 29°C or 18°C.

### Wing preparations and dissection of late pupa

For wing preparations, 1 to 3 day old flies were anesthetized and placed in 95% ethanol. Wings were dissected in ethanol with forceps, transferred to 100% isopropanol on a glass slide, dried briefly, and covered with a drop of Euparal mountant (Fischer Scientific). Pupae of the proper genotype were identified using GFP marked balancer selection and stuck to a slide with double-sided tape. The pupal case was carefully cut away using forceps.

### Wounding assay

The wounding assay was performed on stage 15 embryos as described [9], except that the nitrogen laser-pumped dye laser was connected to an Andor Revolution spinning disc confocal microscope (Andor Technology).

### In situ hybridization and immunochemistry

Standard protocol for *in vitro* transcription of DIG labeled mRNA (using DIG RNA labeling mix, Roche) was followed using linearized *holn1* cDNA (clone GH13760, DGRC) as a template. Whole-mount in situ hybridization was carried out using standard methods [41].

For whole-mount immunochemistry, stage 15 embryos were fixed and stained essentially as described [9], except that fixation was performed in 4% formaldehyde in phosphate buffer (PBS) and blocking was performed over night. We used the following primary antibodies: mouse anti-armadillo 1:50 (Developmental Studies Hybridoma Bank), and rabbit anti-dpERK 1:100 (Santa Cruz Biotechnology). Secondary antibodies and probes used were: Alexa anti-rabbit 488 1:250, Alexa anti-mouse 568, Alexa 594 Phalloidin 1:200 (Invitrogen), and DAPI 1 µg/ml (Sigma-Aldrich).

### Confocal imaging

For live imaging, embryos were collected as described [9], and mounted on their ventral side on glass bottom culture dishes (MatTek Corporation; USA) coated with double-sided tape, on Halocarbon carbon oil 700 (Sigma-Aldrich). Stage 15 live embryos were wounded as described above. The laser power used to wound control versus *holn1* mutant embryos for live imaging was lower than the one used for 16 hour after wounding observation, in order to inflict smaller wounds that would close during imaging procedure. Imaging was performed at 25°C using an Andor Revolution spinning disc confocal microscope (Andor Technology). Individual Z-slices with a step size of 1 µm were taken every 1 minute for 4 hours. For imaging of Ddc-GFP and Msn-DsRed reporters [5], [8], embryos were allowed to develop 5 to 5.5 hours at 25°C after wounding. Percentage of embryos showing wound reporter activation was quantified as previously described [5]. Stained and wound reporter embryos were imaged using a Zeiss LSM 510 Meta or Zeiss LSM 710 confocal microscope, and scanned with 1 µm between slices. All images shown are Z-projections, except stated otherwise, and processed using ImageJ (NIH) and Photoshop (Adobe).

### Scanning Electron Microscopy

Flies were anesthetized and frozen at −20°C for 30 minutes or −80°C for days. Fly heads were removed with a razor blade and mounted on a metal thumb-tac coated with nailpolish. Samples were then sputter coated with gold using a JEOL JFC-1200 machine and imaged within a few days on the Scanning Electron Microscope JEOL JSM-5200LV at 150 or 200X. Images were processed using Photoshop (Adobe).

## Supporting Information

Figure S1 **Percentage of dead embryos observed after wounding.** The graph shows the percentage of dead embryos (unhatched larvae) observed 16 hours post wounding. *holn1^LL07287^* homozygous show significantly higher percentage of dead embryos when compared to other genotypes, including *holn1^LL07287^* heterozygotes and *holn1^c07150^/holn1^LL07287^* transheterozygotes. Fisher's exact test showed significant different between groups (*<0.05**, p<0.01; ***, p<0.0001).
(TIF)

Movie S1 **Time-lapse showing wound closure process in control** ***e22c>cherry-moesin*** **embryo depicted in** **Figure 3A** **.** The first time point (t = 0min) was taken just before wounding. Actin cable formation starts around 10 minutes after wounding and appears to be completely formed and continuous before 20 minutes after wounding. The wound is closed at 110 minutes after wounding. Arrows point towards membrane protrusions. Images were taken in a spinning disc confocal microscope. Images are Z projections of 23 slices (total = 23 µm) scanned at 1-minute intervals. Scale bar = 20 µm.
(AVI)

Movie S2 **Time-lapse showing wound closure process in the** ***e22c>cherry-moesin,holn1^c07150^*** **mutant embryo depicted in** **Figure 3B**. The first time point (t = 0 min) was taken just before wounding. Actin cable formation starts around 15 minutes after wounding and appears to be completely formed and continuous before 30 minutes after wounding. The wound is closed at 200 minutes after wounding. Arrows point towards membrane protrusions. Images were taken in a spinning disc confocal microscope. Images are Z projections of 23 slices (total = 23 µm) scanned at 1-minute intervals. Scale bar = 20 µm.
(AVI)
