# Supplementary figures and images for: Hole-in-One Mutant Phenotypes Link EGFR/ERK Signaling to Epithelial Tissue Repair in Drosophila
Source: PLoS One. 2011 Nov 29;6(11):e28349. doi: 10.1371/journal.pone.0028349 (PMC3226689; doi:10.1371/journal.pone.0028349)

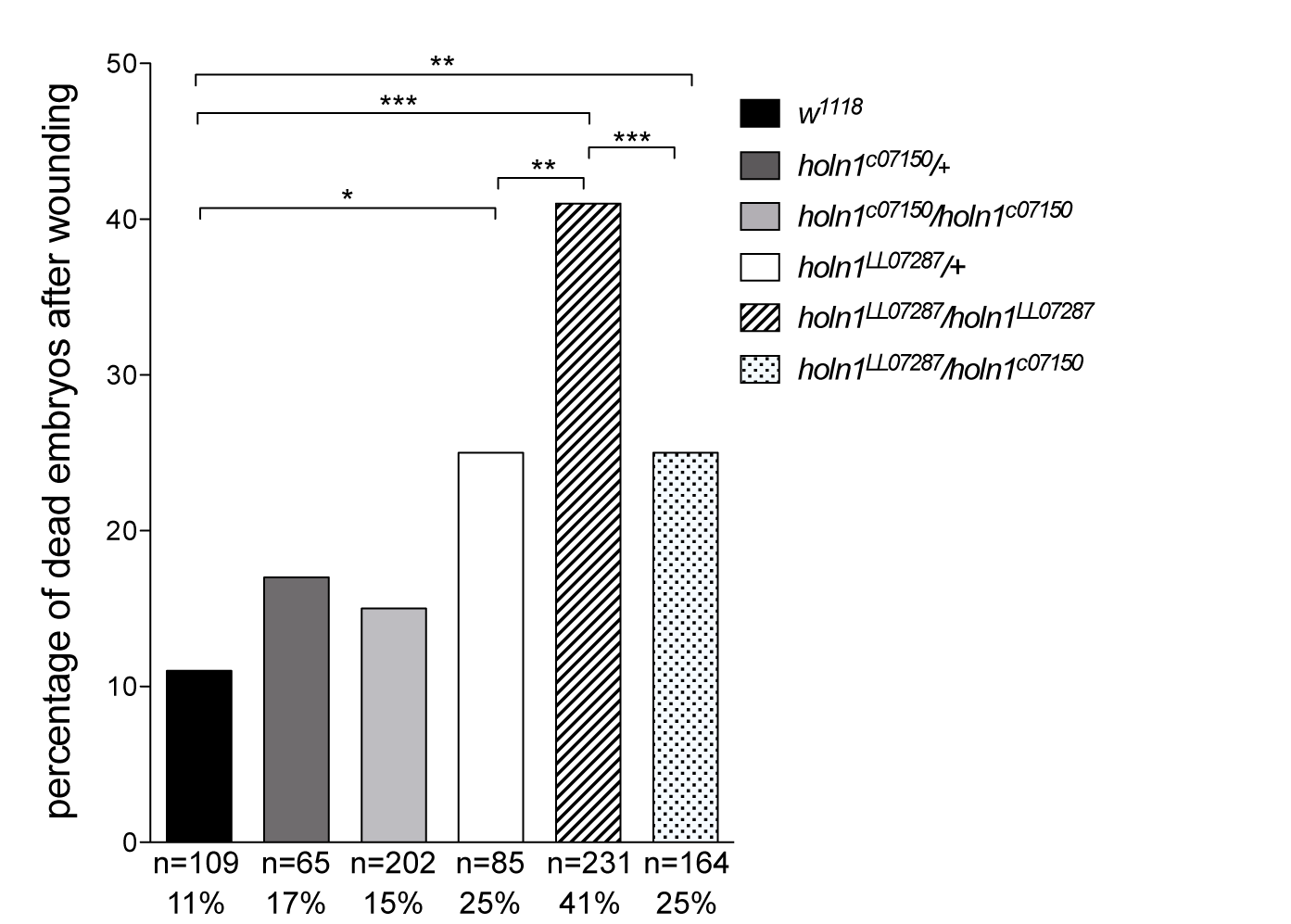

Supplement: Figure S1 — Percentage of dead embryos observed after wounding. The graph shows the percentage of dead embryos (unhatched larvae) observed 16 hours post wounding. holn1LL07287 homozygous show significantly higher percentage of dead embryos when compared to other genotypes, including holn1LL07287 heterozygotes and holn1c07150/holn1LL07287 transheterozygotes. Fisher's exact test showed significant different between groups (*<0.05**, p<0.01; ***, p<0.0001). (TIF) [file pone.0028349.s001.tif]
